# Trends and burden of diabetes in pregnancy among Aboriginal and non-Aboriginal mothers in Western Australia, 1998–2015

**DOI:** 10.1186/s12889-022-12663-6

**Published:** 2022-02-09

**Authors:** Marwan Awad Ahmed, Helen D. Bailey, Gavin Pereira, Scott W. White, Kingsley Wong, Carrington C. J. Shepherd

**Affiliations:** 1 Telethon Kids Institute, University of Western Australia, P.O. Box 855, West Perth, Western Australia 6872 Australia; 2 School of Population and Global Health, The University of Western Australia, Perth, Australia; 3 Curtin School of Population Health, Curtin University, Perth, Western Australia Australia; 4 Centre for Fertility and Health (CeFH), Norwegian Institute of Public Health, Oslo, Norway; 5 Division of Obstetrics and Gynaecology, The University of Western Australia, Perth, WA Australia; 6 Maternal Fetal Medicine Service, King Edward Memorial Hospital, Subiaco, WA Australia; 7 Curtin Medical School, Faculty of Health Sciences, Curtin University, Perth, Australia; 8 Ngangk Yira Research Centre, Murdoch University, Perth, WA Australia

**Keywords:** Aboriginal, Torres Strait islanders, Indigenous, Diabetes in pregnancy, Pre-gestational diabetes, Gestational diabetes, Large for gestational age

## Abstract

**Background:**

Diabetes in pregnancy (DIP), which includes pre-gestational and gestational diabetes, is more prevalent among Aboriginal women. DIP and its adverse neonatal outcomes are associated with diabetes and cardiovascular disease in the offspring. This study investigated the impact of DIP on trends of large for gestational age (LGA) in Aboriginal and non-Aboriginal populations, and added to the limited evidence on temporal trends of DIP burden in these populations.

**Methods:**

We conducted a retrospective cohort study that included all births in Western Australia between 1998 and 2015 using linked population health datasets. Time trends of age-standardised and crude rates of pre-gestational and gestational diabetes were estimated in Aboriginal and non-Aboriginal mothers. Mixed-effects multivariable logistic regression was used to estimate the association between DIP and population LGA trends over time.

**Results:**

Over the study period, there were 526,319 births in Western Australia, of which 6.4% were to Aboriginal mothers. The age-standardised annual rates of pre-gestational diabetes among Aboriginal mothers rose from 4.3% in 1998 to 5.4% in 2015 and remained below 1% in non-Aboriginal women. The comparable rates for gestational diabetes increased from 6.7 to 11.5% over the study period in Aboriginal women, and from 3.5 to 10.2% among non-Aboriginal mothers. LGA rates in Aboriginal babies remained high with inconsistent and no improvement in pregnancies complicated by gestational diabetes and pre-gestational diabetes, respectively. Regression analyses showed that DIP explained a large part of the increasing LGA rates over time in Aboriginal babies.

**Conclusions:**

There has been a substantial increase in the burden of pre-gestational diabetes (Aboriginal women) and gestational diabetes (Aboriginal and non-Aboriginal) in recent decades. DIP appears to substantially contribute to increasing trends in LGA among Aboriginal babies.

**Supplementary Information:**

The online version contains supplementary material available at 10.1186/s12889-022-12663-6.

## Background

It is well-established that diabetes in pregnancy (DIP) poses substantial risks to the fetus/neonate, including high or low birth weight, preterm birth, congenital anomalies and perinatal death [1]. DIP, which includes pre-gestational diabetes mellitus (PGDM) and gestational diabetes mellitus (GDM), also has long-term implications on the offspring. Intrauterine hyperglycemia and its associated adverse neonatal outcomes (high or low birth weight) significantly increase the risk of diabetes and cardiovascular disease in adulthood [2, 3].

The prevalence of DIP is disproportionately high in Indigenous populations worldwide [4]. Among Australian Aboriginal and Torres Strait Islander (the First Nations Peoples of Australia; the term ‘Aboriginal’ is respectfully used hereafter—see *Terminology* in the Methods section for further details) mothers, DIP is more prevalent and the rates of type 2 diabetes mellitus (T2DM) in pregnancy may be ten times higher compared with non-Aboriginal women [5]. The current PGDM rates among Aboriginal women in the Northern Territory of Australia are among the highest in the world [6]. The risk of GDM is 1.5 times higher in Aboriginal mothers [5]. The burden of DIP extends to neonates, as babies born to Aboriginal mothers with diabetes are at higher risk of adverse outcomes [7].

DIP is believed to add, directly or indirectly, to the burden of diabetes in Indigenous populations. Both DIP and high birth weight (HBW) exert a differential impact on the future risk of diabetes in Indigenous compared with non-Indigenous people [8, 9]. Osgood et al. reported that intrauterine exposure to GDM is a major contributor to the intergenerational cycle that leads to the epidemic of type 2 diabetes (T2DM) among Canadian First Nations populations [8]. They concluded that GDM may be responsible for 19 to 30% of T2DM cases among Indigenous people, compared with 6% of cases in non-Indigenous individuals [8]. Indigenous Canadian women who had a HBW had substantially heightened risk of future T2DM and GDM when compared with their non-Indigenous counterparts [9].

The increasing rate of DIP has been a global trend [10–12]. In Australia, there is limited evidence on the change in the burden of DIP overtime among Aboriginal mothers. There is also scarcity of evidence about the temporal contribution of DIP to population-wide adverse neonatal outcomes and the possible long-term effect of DIP on Aboriginal offspring. A recent study from Australia’s Northern Territory revealed that, over 30 years, the prevalence of PGDM increased ten-fold among Aboriginal mothers [6]. The study also found that DIP largely explained the increasing rates of LGA among Aboriginal women over three decades [6]. Evidence from other culturally distinct Aboriginal populations in other Australian states is lacking.

Studies incorporating the dimension of time provide a better understanding of the burden of DIP and its adverse perinatal outcomes on the intergenerational and long-term risk of chronic disease in the Aboriginal population. The established link between the disproportionate burden of cardiometabolic disease among Aboriginal people and their rapid shift toward a more sedentary lifestyle and less healthy diet [13, 14] adds to the importance of temporal shifts in studying such disparities. Investigating the interaction of DIP and its complications with time, besides describing the patterns of DIP-related inequalities, can shed light on possible distal drivers of health disparities between Aboriginal and non-Aboriginal people.

This study aims to compare the time trends of the burden of DIP in Aboriginal and non-Aboriginal populations, and to investigate whether, and to what extent, this burden impacts on trends of large for gestational age (LGA, as a measure of accelerated fetal growth that takes sex and gestational age into account) births in these populations.

## Methods

### Study design and data sources

This retrospective cohort study used population health datasets linked by the Data Linkage Branch (DLB) of the Western Australia Government Department of Health. DLB uses probabilistic matching methods to link datasets and provides researchers with de-identified data via secure data transfer methods [15] . Datasets linked include the Midwives Notification System (MNS), Hospital Morbidity Database Collection (HMDC), Births Registrations and Death Registrations. MNS, our primary data source contains details on the circumstances of all births occurring at ≥20 weeks’ gestation or birth weight of 400 g or more in Western Australia. HMDC includes information about inpatient episodes in all Western Australian private and public hospitals.

### Study population

The study included all births at ≥20 weeks’ gestation occurring in WA between 1st January 1998 and 31st December 2015. A multiple pregnancy was counted once when calculating DIP rates. Multiple births (*n* = 15,558) were excluded from other analyses. Records missing GDM (*n* = 1912, 0.4%) and PGDM (*n* = 2056, 0.4%) were excluded from descriptive analyses. Multivariable analyses of LGA trends excluded records with missing gestational age (*n* = 2177, 0.4%) and birth weight (*n* = 103, < 0.1%). While remoteness and socioeconomic status were missing in 8232 (1.6%) and 21,443 (4.1%) records, respectively, the percentage of missing values for all other variables was below 0.5%.

### Terminology

The term ‘Aboriginal’ is respectfully used throughout this paper to refer to the First Nations peoples of the Australian continent—Aboriginal and Torres Strait Islander peoples. The term is used for the purpose of brevity and in preference to ‘Indigenous’. While ‘Aboriginal’ is often the preferred term in Western Australian settings (and is a more specific term than ‘Indigenous’), we recognise that it is a generic term that excludes any description of language group or Country and that it is not the preferred term among all Aboriginal and Torres Strait Islander peoples.

### Data management

#### Exposure

We used the Indigenous status flag introduced by the Western Australian Data Linkage Branch in 2014 to support a more complete and consistent identification of Aboriginal peoples in administrative data. This flag is created from a number of Western Australian core datasets using an algorithm created by the ‘Getting our Story Right’ project [16]. The project used a multi-stage median approach to generate a single Aboriginal status for each individual. PGDM was ascertained from the MNS and HMDC as a principal or additional diagnosis using the ICD-9-AM code 250 and ICD-10-AM codes E10–11, E13–14 and O24.0–24.3. Births of the same mother were linked within the MNS by the unique maternal identifier, allowing us to ascertain PGDM in earlier pregnancies. GDM was ascertained from the MNS and from HMDC as a principal or additional diagnosis using the relevant ICD codes (ICD-9-AM code 648.8 and ICD-10-AM codes O24.4, O24.9).

#### Outcomes and covariates

Using Australian sex- and gestational age-specific birth weight percentiles [17], LGA was defined as birth weight >90th percentile and SGA as birth weight < 10th percentile. Perinatal death was defined as stillbirth (fetal death at gestational age ≥ 20 weeks or with ≥400 g birth weight) or neonatal death (death during the first 28 days of life). Preterm birth was defined as birth before 37 weeks’ gestation. Based on the mother’s residence, remoteness was classified into two categories (Remote and Very remote residence, or other) according to the Accessibility/Remoteness Index of Australia (ARIA) [18]. The values of the area-based Index of Relative Socio-economic Disadvantage of the mother’s residence at the birth was derived from the Census closest to the birth year and categorised into tertiles [19].

### Statistical analysis

Characteristics of mothers with PGDM and GDM, stratified by Aboriginal status and six-year birth periods (1998–2003, 2004–2009 and 2010–2015), were described using counts and proportions. P trend was used to assess the statistical significance of linear trends in neonatal outcomes and maternal characteristics over the three periods. P trend relies on calculating a chi-square statistic for the linear trend (regression) of the proportion of the characteristic (as the outcome variable) on the time periods (as the explanatory variable) [20, 21].

We calculated age-standardised rates for PGDM and GDM to account for the vastly different age profile of Aboriginal and non-Aboriginal mothers. Direct age-standardisation was performed using all mothers who gave birth in WA during the study period as the standard population.

Mixed-effects multivariable logistic regression models, with a random intercept for mother to account for the clustering effect from births to the same mother, were used to examine the contribution of DIP to the trends of LGA in Aboriginal and non-Aboriginal populations by estimating odds ratios (OR) and 95% confidence intervals (CIs). The effects of adding PGDM, GDM and then both types of diabetes to an adjusted model that had LGA as the dependent variable and year (year of birth divided by 10, in a continuous form, so that the change in OR can be interpreted as change per decade) as the independent variable were studied. To assess the assumption of linearity between log odds LGA and year, we applied locally weighted regression (*lowess* in Stata). Factors known to be associated with birth weight were initially considered for inclusion as covariates. We then excluded variables not associated with the primary independent variable of interest (year of birth) from the models. The covariates included in the models were maternal age (continuous), remoteness (binary), parity group (categorical: 0, 1, 2 and 3 or more), and smoking during pregnancy (binary). All analyses were conducted using Stata version 16 (StataCorp. 2019).

## Results

There were 526,319 births to 299,158 mothers in Western Australia between 1998 and 2015. About 6.4% of births (*n* = 33,696) were to Aboriginal mothers. There were distinct differences in most demographic and other characteristics by Aboriginal status, in both pregnancies complicated by DIP (Tables 1 and 2) and all pregnancies (Additional file 1: Table S1). Notably, Aboriginal mothers were younger, had a higher parity, and were more likely to live in a low SES or remote/very remote area, and smoke during pregnancy.

**Table 1:** Maternal and neonatal characteristics of Aboriginal and non-Aboriginal singleton pregnancies complicated by pre-gestational diabetes, 1998–2015

Characteristic	Aboriginal Mothers				Non-Aboriginal mothers			
	1998 to 2003 (*n* = 266)	2004 to 2009 (*n* = 293)	2010 to 2015 (*n* = 354)	*P trend*	1998 to 2003 (*n* = 730)	2004 to 2009 (*n* = 1096)	2010 to 2015 (*n* = 1546)	*P trend*
**Maternal Characteristics**								
Maternal age (years)								
25 or below	75 (28.2)	66 (22.5)	71 (20.1)	0.019	146 (20.0)	154 (14.1)	194 (12.5)	< 0.001
> 25 to 35	138 (51.9)	152 (51.9)	215 (60.7)	0.021	443 (60.7)	681 (62.1)	956 (61.8)	0.663
above 35	53 (19.9)	75 (25.6)	68 (19.2)	0.702	141 (19.3)	261 (23.8)	396 (25.6)	0.001
Mean (SD)^a^	29.6 (6.4)	30.8 (6.3)	30.6 (6.1)	0.096	30.4 (5.6)	31.6 (5.4)	31.8 (5.3)	< 0.001
Parity group								
0	36 (13.5)	36 (12.3)	57 (16.1)	0.320	248 (34.0)	334 (30.5)	524 (34.0)	0.671
1	46 (17.3)	43 (14.7)	72 (20.3)	0.266	260 (35.6)	430 (39.2)	542 (35.1)	0.486
2	56 (21.1)	47 (16.0)	70 (19.8)	0.783	145 (19.9)	198 (18.1)	282 (18.3)	0.435
3 plus	128 (48.1)	167 (57.0)	155 (43.8)	0.186	77 (10.5)	134 (12.2)	195 (12.6)	0.177
Caesarean delivery	125 (47.0)	171 (58.4)	216 (61.0)	0.001	397 (54.5)	672 (61.3)	890 (58.0)	0.295
Smoking during pregnancy	119 (44.7)	153 (52.2)	186 (52.5)	0.065	151 (20.7)	161 (14.7)	166 (10.8)	< 0.001
SES tertiles								
1st (most disadvantaged)	196 (85.2)	214 (81.4)	265 (82.0)	0.368	279 (39.9)	385 (36.2)	544 (36.4)	0.167
2nd	< 35 (< 15.2)^b^	39 (14.8)	40 (12.4)	0.760	233 (33.3)	375 (35.2)	522 (34.9)	0.536
3rd (least disadvantaged)	< 10 (< 4.3)^b^	10 (3.8)	18 (5.6)	0.022	187 (26.8)	304 (28.6)	428 (28.6)	0.407
Remote or very remote residence	155 (63.3)	178 (62.5)	213 (60.7)	0.512	100 (13.9)	109 (10.1)	70 (4.6)	< 0.001
Preterm birth	82 (30.9)	107 (36.5)	128 (36.3)	0.191	192 (26.3)	289 (26.4)	391 (25.5)	0.617
Preeclampsia	50 (18.8)	28 (9.6)	27 (7.6)	< 0.001	131 (18.0)	73 (6.7)	106 (6.9)	< 0.001
**Neonatal Characteristics**								
Perinatal death	14 (5.3)	11 (3.8)	15 (4.2)	0.573	15 (2.1)	15 (1.4)	16 (1.0)	0.055
Female sex	124 (46.6)	148 (50.5)	175 (49.4)	0.522	356 (48.8)	532 (48.5)	751 (48.6)	0.943
LGA	89 (33.6)	100 (34.1)	116 (32.9)	0.833	280 (38.4)	423 (38.6)	502 (32.7)	0.002
SGA	33 (12.5)	26 (8.9)	29 (8.2)	0.087	30 (4.1)	46 (4.2)	71 (4.6)	0.533

Data represented as numbers (percentages)

*LGA* large for gestational age, *SD* standard deviation, *SES* socio-economic status, *SGA* small for gestational age

^a^The over time trends of maternal age (as a continuous variable) were assessed by simple linear regression

^b^To maintain confidentiality, the numbers (percentages) are not shown for small cells. Numbers in the second least prevalent groups are also not shown to prevent calculating numbers in small cells

**Table 2:** Maternal and neonatal characteristics of Aboriginal and non-Aboriginal singleton pregnancies complicated by gestational diabetes, 1998–2015

Characteristic	Aboriginal Mothers				Non-Aboriginal mothers			
	1998 to 2003 (*n* = 404)	2004 to 2009 (*n* = 539)	2010 to 2015 (*n* = 917)	*P trend*	1998 to 2003 (*n* = 5088)	2004 to 2009 (*n* = 7362)	2010 to 2015 (n = 15,447)	*P trend*
**Maternal Characteristics**								
Maternal age (years)								
25 or below	140 (34.7)	192 (35.6)	356 (38.8)	0.114	590 (11.6)	855 (11.6)	1657 (10.7)	0.037
> 25 to 35	210 (52.0)	274 (50.8)	425 (46.3)	0.038	3249 (63.9)	4484 (60.9)	9896 (64.1)	0.106
above 35	54 (13.4)	73 (13.5)	136 (14.8)	0.429	1249 (24.5)	2023 (27.5)	3894 (25.2)	0.777
Mean (SD)^a^	28.2 (6.2)	28.1 (6.2)	28.1 (6.3)	0.609	31.7 (5.3)	32.1 (5.3)	31.9 (5.2)	0.145
Parity group								
0	70 (17.3)	98 (18.2)	238 (26.0)	< 0.001	1938 (38.1)	3098 (42.1)	6814 (44.3)	< 0.001
1	81 (20.0)	108 (20.0)	208 (22.7)	0.210	1731 (34.0)	2374 (32.2)	5056 (32.9)	0.314
2	76 (18.8)	96 (17.8)	163 (17.8)	0.690	856 (16.8)	1099 (14.9)	2179 (14.2)	< 0.001
3 plus	177 (43.8)	237 (44.0)	307 (33.5)	< 0.001	563 (11.1)	791 (10.7)	1322 (8.6)	< 0.001
Caesarean delivery	141 (34.9)	188 (34.9)	356 (38.9)	0.108	1882 (37.0)	3161 (42.9)	6545 (42.7)	< 0.001
Smoking during pregnancy	166 (41.1)	208 (38.6)	373 (40.8)	0.926	814 (16.0)	818 (11.1)	1084 (7.1)	< 0.001
SES tertiles								
1st (most disadvantaged)	259 (73.0)	368 (75.4)	627 (72.0)	0.512	1709 (35.1)	2498 (35.0)	5357 (35.6)	0.385
2nd	77 (21.7)	95 (19.5)	188 (21.6)	0.844	1692 (34.8)	2383 (33.4)	5173 (34.4)	0.941
3rd (least disadvantaged)	19 (5.4)	25 (5.1)	56 (6.4)	0.369	1465 (30.1)	2257 (31.6)	4498 (29.9)	0.328
Remote or very remote residence	204 (54.3)	249 (47.8)	360 (39.4)	< 0.001	428 (8.6)	601 (8.3)	557 (3.6)	< 0.001
Preterm birth	51 (12.6)	69 (12.8)	133 (14.5)	0.292	464 (9.1)	727 (9.9)	1454 (9.5)	0.687
Preeclampsia	42 (10.4)	37 (6.9)	35 (3.8)	< 0.001	433 (8.5)	317 (4.3)	384 (2.5)	< 0.001
**Neonatal Characteristics**								
Perinatal death	< 10 (< 2.5)^b^	< 10 (< 1.9)^b^	11 (1.2)	0.432	27 (0.5)	33 (0.4)	40 (0.3)	0.002
Female sex	204 (50.5)	251 (46.6)	418 (45.6)	0.118	2418 (47.5)	3468 (47.1)	7463 (48.3)	0.172
LGA	120 (29.7)	118 (21.9)	193 (21.1)	0.002	978 (19.2)	1259 (17.1)	2029 (13.3)	< 0.001
SGA	32 (7.9)	42 (7.8)	65 (7.1)	0.563	310 (6.1)	507 (6.9)	1277 (8.3)	< 0.001

Data represented as numbers (percentages)

*LGA* large for gestational age, *SD* standard deviation, *SES* socio-economic status, *SGA* small for gestational age

^a^The over time trends of maternal age (as a continuous variable) were assessed by simple linear regression

^b^To maintain confidentiality, the numbers (percentages) are not shown for small cells

The overall age-standardised prevalence of PGDM was 4.6% (95% CI: 4.3, 4.9) in Aboriginal women and 0.7% (95% CI: 0.68, 0.72) among their non-Aboriginal counterparts. The age-standardised prevalence of GDM was 7.9% (95% CI: 7.6, 8.3) and 5.8% (95% CI: 5.7, 5.9) in Aboriginal and non-Aboriginal mothers, respectively.

In pregnancies complicated by PGDM (Table 1), LGA rates among births to Aboriginal women remained stable over time (approximately one-third of these births) and decreased considerably in non-Aboriginal births (from 38.4 to 32.7%). Smoking rates among Aboriginal mothers with PGDM increased by about eight percentage points, to 52.5% by 2010–15. For non-Aboriginal mothers, on the other hand, there was a consistent drop in smoking rates over the study period (from 20.7 to 10.8%). The rates of preeclampsia decreased with time in both Aboriginal and non-Aboriginal mothers.

In GDM pregnancies (Table 2), there was a decrease in LGA rates among births to Aboriginal and non-Aboriginal mothers over the three time periods (29.7, 21.9 and 21.1% in Aboriginal and 19.2, 17.1, 13.3% in non-Aboriginal babies—in 1998–2003, 2004–09 and 2010–15, respectively). Smoking rates remained high (around 40%) in Aboriginal pregnancies complicated by GDM with no marked improvement over time. In contrast, there was a consistent (and substantial) decrease in smoking rates among non-Aboriginal mothers, to 7.1% in 2010–15. As with PGDM, preeclampsia rates decreased in Aboriginal and non-Aboriginal pregnancies complicated by GDM.

### Trends in pre-gestational diabetes

**Fig. 1:**
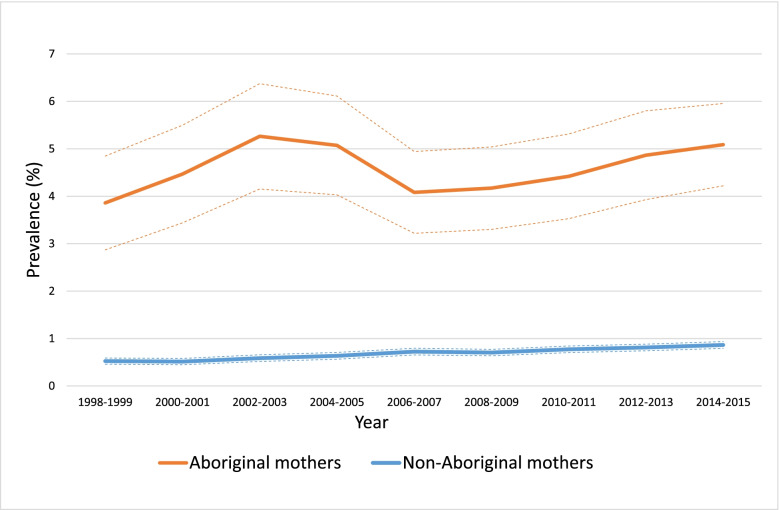
Age-standardised prevalence of pre-gestational diabetes among Aboriginal and non-Aboriginal pregnancies, 1998–2015. The dotted lines are the 95% confidence intervals

There was both a substantially higher prevalence and more rapid rates of change in PGDM over time among Aboriginal women (Fig. 1 and Additional file 2: Fig. S1). Between 1998 and 2015, the age-standardised prevalence rates of PGDM grew from 4.3% (95% CI: 2.7, 5.9) to 5.4% (95% CI: 4.1, 6.7) (Fig. 1) and the crude rates increased from 2.3% (95% CI: 1.7, 3.1) to 3.8% (95% CI: 3.1, 4.8) (Additional file 2: Fig. S1) among Aboriginal mothers. In non-Aboriginal women, age-standardised and crude annual rates of PGDM were similar, and increased from 0.6% (95% CI: 0.5, 0.7) in 1998 to 0.9% (95% CI: 0.8, 1.0) in 2015.

### Trends in gestational diabetes

**Fig. 2:**
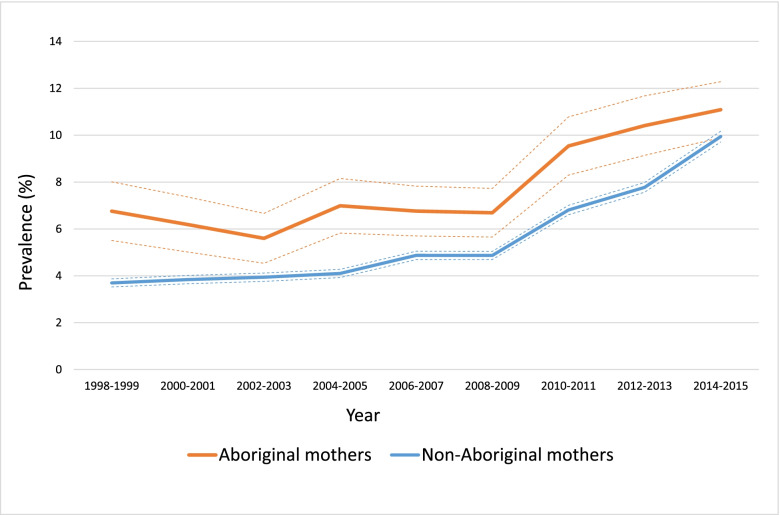
Age-standardised prevalence of gestational diabetes among Aboriginal and non-Aboriginal pregnancies, 1998–2015. The dotted lines are the 95% confidence intervals

The age-standardised rates of GDM were consistently higher in Aboriginal women during the study period (Fig. 2), although the trends over time in both standardised and crude rates (Additional file 3: Fig. S2) were comparable. Among Aboriginal mothers, the age-standardised rates of GDM increased from 6.7% (95% CI: 4.8, 8.5) to 11.5% (95% CI: 9.7 to 13.3) and the crude rates increased from 4.6% (95% CI: 3.7, 5.7) to 9.1% (95% CI: 7.9, 10.4) between 1998 and 2015. Among non-Aboriginal mothers, the age-standardised rates of GDM increased from 3.5% (95% CI: 3.3, 3.8) to 10.2% (95% CI: 9.9, 10.6) and the crude rates increased from 3.4% (95% CI: 3.2, 3.7) to 10.6% (95% CI: 10.3, 11.0) over this period.

### Impact of diabetes in pregnancy on LGA trends in population

Our initial mixed-effects multivariable model estimated a 16% increase per decade in the odds of LGA among births to Aboriginal women (OR = 1.16, 95% CI: 1.04, 1.29) after adjusting for maternal age, parity, remoteness and smoking during pregnancy, as well as for clustering by mother (Table 3). The elevated odds was attenuated to 10% by further adjustment for GDM (OR = 1.10, 95% CI: 0.99, 1.23) and to 14% by accounting separately for PGDM (OR = 1.14, 95% CI: 1.02, 1.26). Simultaneous adjustment for both GDM and PGDM resulted in a 7% increase per decade (OR = 1.07, 95% CI: 0.97 to 1.19). Among non-Aboriginal mothers, the odds of LGA per decade decreased by 6% (OR = 0.94, 95% CI: 0.92, 0.97) after adjusting for the same variables in the initial model above. The magnitude of the decrease was 7% (OR = 0.93, 95% CI: 0.91, 0.95) and 6% (OR = 0.94, 95% CI: 0.91, 0.96) after accounting for GDM and PGDM, respectively. Compared to the initial model, adjusting for both GDM and PGDM reduced LGA odds by two percentage points and resulted in 8% overall decrease in the odds of LGA per decade (OR = 0.92, 95% CI: 0.90, 0.94).

**Table 3:** Mixed-effects logistic regression for the association between LGA and time (per decade), stratified by Aboriginal status

	Initial Model^a^	Added to Initial Model		
		Gestational diabetes	Pre-gestational diabetes	Both gestational and pre-gestational diabetes
	OR (95% CI)	OR (95% CI)	OR (95% CI)	OR (95% CI)
Aboriginal mothers	1.16 (1.04 to 1.29)	1.10 (0.99 to 1.23)	1.14 (1.02 to 1.26)	1.07 (0.97 to 1.19)
Non-Aboriginal mothers	0.94 (0.92 to 0.97)	0.93 (0.91 to 0.95)	0.94 (0.91 to 0.96)	0.92 (0.90 to 0.94)

*CI* Confidence intervals, *LGA* large for gestational age, *OR* Odds ratio

^a^Adjusted for maternal age, parity, remoteness and smoking

The supplementary figure (Additional file 4: Fig. S3) shows that the crude rates of LGA exhibited an increase in the total Aboriginal population and a slight decrease in the non-Aboriginal population over the study period.

## Discussion

### Trends of pre-gestational diabetes

In this study, the annual age-standardised and crude rates of PGDM grew considerably (to 5.4 and 3.8%, respectively) in the last two decades among Aboriginal mothers but remained below 1% in non-Aboriginal mothers. This is consistent with the extant evidence-base—which confirms the relatively heavy burden of PGDM in Indigenous populations at national [5, 22, 23] and global [24–26] levels—with the highest prevalence rates of PGDM reported among Aboriginal mothers in Central Australia (8.4%) [6], while PGDM complicated 6.3% of pregnancies among Arizona’s Pima population in the United States [27].

The higher prevalence of PGDM in Indigenous populations is a reflection of the elevated rates (and contrasting demographic characteristics) of T2DM [28]. T2DM accounts for more than 95% of PGDM cases among Australian Aboriginal mothers compared to approximately 55% in non-Aboriginal women [6, 29]. In 2015, the prevalence of T2DM (which is preventable) in pregnancy was approximately eight times higher in Western Australian Aboriginal mothers compared with their non-Aboriginal counterparts [29]. Disproportionately higher rates of T2DM in Aboriginal youth [30, 31] and the differentially higher incidence of T2DM among Aboriginal females [32, 33] contribute to the disparate burden of PGDM in this population.

Our observation of steeper increasing trends in Indigenous populations (relative to their respective general populations) is a feature of the global health disparities literature [34]. Hare et al., for example, recently reported a ten-fold increase in PGDM among Aboriginal mothers over 30 years in Australia’s Northern Territory, with a crude prevalence of 5.7% in 2016 [6]. They found that PGDM rates remained ≤0.7% through the study period in non-Aboriginal mothers. The reasons for the sharp growth in the burden of T2DM among Indigenous populations are complex and multifaceted—although at least in part reflect the rapid behavioural and environmental changes towards urban, sedentary lifestyle and energy-dense diets [13, 14] and the concomitant longer-term legacies of colonisation that have given rise to social and economic circumstances that shape the affordability and accessibility of nutritious foods, healthcare and other proximal determinants of chronic diseases. The steeper increase in PGDM in the Northern Territory study compared to ours may reflect demographic circumstances, whereby a larger proportion of Aboriginal people in the Northern Territory live in remote areas [35] where a change in lifestyle may be more impactful on life outcomes.

### Trends of gestational diabetes

Our results showed that the annual age-standardised rates of GDM increased consistently in Aboriginal mothers and remained higher than that reported in their non-Aboriginal counterparts over the two decades to 2015. Annual crude rates of GDM were comparable in Aboriginal and non-Aboriginal mothers. The discrepancies in age structure between Aboriginal and non-Aboriginal mothers, and the established link between the risk of GDM and maternal age [36] indicate that age-standardised rates offer a more meaningful comparison. Crude rates underestimate the disparities between the two populations.

There is a worldwide increasing trends in the prevalence of GDM [37–39] that is believed to be explained by an increase in maternal obesity and older maternal age at birth [40, 41]. Temporal increases in GDM have also been reported in Australian Aboriginal mothers [42]. The crude prevalence increased from 3.4 to 13% between 1987 and 2016 in the Northern Territory of Australia [6]. There has been evidence from population-level studies of both increasing and stable rates of GDM in Canadian First Nations mothers [34, 43].

Interpreting prevalence trends of GDM is not straightforward and should be considered in conjunction with the temporal changes in screening policies and diagnostic criteria. In 2013, the Australasian Diabetes in Pregnancy Society (ADIPS) adopted the 2010 recommendations of the International Association of Diabetes and Pregnancy Study Groups (Additional file 5: Table S2) [44]. The implementation of the new guidelines was associated with a substantial increase in the prevalence of GDM globally [45], and we expect a similar contribution to the increasing trends of GDM among both Aboriginal and non-Aboriginal pregnant women in Western Australia. Moreover, between and within jurisdiction discrepancies in the application of diagnostic and screening guidelines [46] may invalidate direct comparisons.

A retrospective audit in rural Western Australia has recently reported inadequate screening for GDM, especially among Aboriginal mothers [47]. Limitations related to the storage of blood samples prior to testing for GDM were also reported in rural and remote Western Australia [48]. Thus, our findings may have underestimated the real prevalence of GDM among the Aboriginal population who are more likely to reside and receive ante-natal care in these areas [29].

### Diabetes in pregnancy and LGA over time

The present study reported that DIP may explain a large part of the increasing trend in LGA among the Aboriginal population. Statistically accounting for PGDM and GDM (separately and collectively) attenuated the LGA increase per decade. Similar results were recently reported among the Aboriginal people of Australia’s Northern Territory, but with a stronger impact of PGDM on LGA trends compared to ours, reflecting a sharper rise of PGDM rates among that Aboriginal population over time in the Northern Territory [6].

In line with the impact of DIP on the overall LGA trends among the Aboriginal population, absolute rates of LGA remained high in Aboriginal babies born to mothers with DIP. LGA continued to affect about one-third of Aboriginal births complicated by PGDM, with no signs of improvement over the study period. Despite the improving trends, LGA continued to impact more than 20% of Aboriginal births complicated by GDM.

### Diabetes in pregnancy, LGA and intergenerational diabetes

The association between exposure to intrauterine hyperglycemia and elevated risk for future diabetes in offspring was reported in Indigenous populations in North America [49–52]. Among Canadian Indigenous women, being born HBW was associated with differentially increased risk of T2DM and GDM, and is believed to play a role in the intergenerational transfer of diabetes risk [9, 53]. Although the present study did not investigate the intergenerational effect of DIP, our findings regarding the over time burden of DIP and its associated LGA are probably in-line with the DIP-driven intergenerational cycle of diabetes in Indigenous populations.

### Health risks may add to burden of diabetes in pregnancy

We reported smoking rates of approximately 40–50% among Aboriginal mothers with GDM and PGDM, with no improvement over the study period. Maternal exposure to tobacco smoke during pregnancy significantly increases the risk of T2DM [54, 55], obesity [56] and GDM [57] in offspring. Epigenetic modifications are believed to mediate these effects [58]. The sizeable rates of maternal smoking may thus add to the intergenerational impact of DIP in the Aboriginal population. Likewise, low birth weight may synergistically interact with genetic susceptibility to obesity to heighten the future risk of T2DM [59]. Given the substantially higher prevalence of many health risks in Aboriginal Australians [60], it is plausible that there may be similar interactions between DIP and health risk behaviours with potential intergenerational implications.

### Positive findings

The present study also reported some positive findings. The rates of pre-eclampsia decreased in Aboriginal mothers with GDM and PGDM, and LGA decreased in Aboriginal pregnancies complicated by GDM. These positive trends can probably be attributed to improved antenatal care [61], increasing awareness about GDM following the *Hyperglycemia and Adverse Pregnancy Outcomes* study [62, 63], increased screening rates and the introduction of new diagnostic criteria [44]. These factors might have resulted in better glycemic control and capturing of the less severe cases of GDM.

### Strengths and limitations of the study

The use of linked, population-level datasets from multiple sources is a strength of the present study. This eliminates selection bias; allows tracking of babies and mothers upon transfer between different levels of health facilities; and maximises the external validity of the study. Utilizing multiple sources of data also increases the sensitivity of capturing medical conditions by enabling the identification of additional cases. Further, we have used a robust, agreed algorithm for the identification of Aboriginal people in the study data sources [16]. This has been shown to reduce missing data and improve within-individual consistency across time, and is likely to have minimised any potential for under-estimation of increasing DIP trends.

Our study does, however, have some limitations. We used routinely collected data that are not originally designed for research purposes. The capture of DIP over time in the HMDC is impacted by changes in the Australian diabetes coding standards that implies modifications to the documentation of diabetes during a hospital admission [64]. However, we believe that using both MNS and HMDC has improved the capture of DIP. Absence of data on maternal weight is also a limitation of this study. Obesity, which is increasing over time in Australia [65], is linked to both DIP and HBW and can be a source of unmeasured temporal confounding that may explain part of the relationship between DIP and LGA [6]. Moreover, we did not have data on glycemic control biomarkers. Hyperglycemia during pregnancy is an established risk factor for adverse fetal outcomes, and its trends might have explained part of the trend in these outcomes.

## Conclusions

Our whole-population study reported a disproportionately growing burden of PGDM and increasing rates of GDM among Aboriginal mothers in Western Australia between 1998 and 2015. DIP appears to explain a high proportion of the increase in LGA trends in the Aboriginal population.

The fact that T2DM, which can be preventable, is behind the vast majority of PGDM cases among the Aboriginal mothers highlights the need for appropriate primary prevention strategies. Increasing GDM trends despite inadequate screening among Aboriginal women points to both the substantial, possibly hidden, size of the problem, the need for improving prenatal care and, concomitantly, the potential for interrupting the impact of the cycle of intergenerational diabetes among the Aboriginal population.

## Supplementary Information

**Additional file 1: Table S1.** Maternal and neonatal characteristics of Aboriginal and non-Aboriginal singleton pregnancies in Western Australia, 1998–2015.

**Additional file 2: Figure S1.** Crude prevalence of pre-gestational diabetes among Aboriginal and non-Aboriginal pregnancies, 1998–2015.

**Additional file 3: Figure S2.** Crude prevalence of gestational diabetes among Aboriginal and non-Aboriginal pregnancies, 1998–2015.

**Additional file 4: Figure S3.** Rates of LGA over time in the Aboriginal and non-Aboriginal populations in Western Australia, 1998–2015.

**Additional file 5: Table S2.** National guidelines for screening and diagnosis of gestational diabetes during the study period (1998–2015).

## Data Availability

Datasets used in this study are not publicly available and cannot be provided by authors due to restrictions by the data custodians. Researchers wanting to access these datasets should refer to the Data Linkage Branch of the Western Australia Government Department of Health (www.datalinkage-wa.org.au).

## Abbreviations

CI: Confidence interval
DIP: Diabetes in pregnancy
GDM: Gestational diabetes mellitus
HBW: High birth weight
HMDC: Hospital Morbidity Database Collection
LGA: Large for gestational age
MNS: Midwives Notification System
OR: Odds ratio
PGDM: Pre-gestational diabetes mellitus
T2DM: Type 2 diabetes mellitus
