# Supplementary material for: Trends and burden of diabetes in pregnancy among Aboriginal and non-Aboriginal mothers in Western Australia, 1998–2015
Source: BMC Public Health. 2022 Feb 9;22:263. doi: 10.1186/s12889-022-12663-6 (PMC8827280; doi:10.1186/s12889-022-12663-6)
Supplement: Supplementary file 1 — Additional file 1: Table S1. Maternal and neonatal characteristics of Aboriginal and non-Aboriginal singleton pregnancies in Western Australia, 1998–2015. [file 12889_2022_12663_MOESM1_ESM.docx]

Table S1: Maternal and neonatal characteristics of Aboriginal and non-Aboriginal singleton pregnancies in Western Australia, 1998-2015

| Characteristic | | Aboriginal mothers | | | | Non-Aboriginal mothers | | | |
| --- | --- | --- | --- | --- | --- | --- | --- | --- | --- |
|  |  | 1998 to 2003 (n=10041) | 2004 to 2009 (n=11228) | 2010 to 2015 (n=11576) | *P trend* | 1998 to 2003 (n=136385) | 2004 to 2009 (n= 156830) | 2010 to 2015 (n= 184701) | *P trend* |
| **Maternal Characteristics** | |  |  |  |  |  |  |  |  |
| Maternal age (years) | |  |  |  |  |  |  |  |  |
|  | 25 or below | 6161 (61.4) | 6834 (60.9) | 6726 (58.1) | <0.001 | 33647 (24.7) | 36102 (23.0) | 36871 (20.0) | <0.001 |
|  | >25 to 35 | 3439 (34.3) | 3753 (33.4) | 4138 (35.8) | 0.017 | 85287 (62.5) | 95491 (60.9) | 117101 (63.4) | <0.001 |
|  | above 35 | 430 (4.3) | 636 (5.7) | 710 (6.1) | <0.001 | 17426 (12.8) | 25215 (16.1) | 30688 (16.6) | <0.001 |
|  | Mean (SD)* | 24.3 (5.8) | 24.5 (6.0) | 25.0 (5.9) | <0.001 | 29.3 (5.5) | 29.8 (5.6) | 30.2 (5.4) | <0.001 |
| Parity group | |  |  |  |  |  |  |  |  |
|  | 0 | 2749 (27.4) | 3433 (30.6) | 3549 (30.7) | <0.001 | 56230 (41.2) | 66344 (42.3) | 79282 (43.1) | <0.001 |
|  | 1 | 2339 (23.3) | 2562 (22.8) | 2912 (25.2) | 0.001 | 47205 (34.6) | 54070 (34.5) | 64450 (35.1) | 0.004 |
|  | 2 | 1781 (17.8) | 1941 (17.3) | 2063 (17.8) | 0.827 | 21381 (15.7) | 23501 (15.0) | 26617 (14.5) | <0.001 |
|  | 3 plus | 3161 (31.5) | 3285 (29.3) | 3036 (26.3) | <0.001 | 11540 (8.5) | 12887 (8.2) | 13410 (7.3) | <0.001 |
| Caesarean delivery | | 1815 (18.1) | 2540 (22.7) | 2984 (25.9) | <0.001 | 36335 (26.7) | 52129 (33.3) | 62774 (34.3) | <0.001 |
| Smoking during pregnancy | | 5111 (51.0) | 5613 (50.1) | 5312 (46.1) | <0.001 | 26026 (19.1) | 21805 (13.9) | 16228 (8.9) | <0.001 |
| SES tertiles | |  |  |  |  |  |  |  |  |
|  | 1st (most disadvantaged) | 7005 (79.1) | 8120 (77.6) | 8170 (73.6) | <0.001 | 44192 (34.3) | 48415 (32.0) | 59023 (32.9) | <0.001 |
|  | 2nd | 1488 (16.8) | 1783 (17.0) | 2177 (19.6) | <0.001 | 44089 (34.2) | 51292 (33.9) | 62159 (34.7) | 0.003 |
|  | 3rd (least disadvantaged) | 358 (4.0) | 563 (5.4) | 753 (6.8) | <0.001 | 40622 (31.5) | 51599 (34.1) | 58151 (32.4) | <0.001 |
| Remote or very remote residence | | 4747 (50.1) | 5169 (47.1) | 4511 (39.2) | <0.001 | 13680 (10.3) | 15175 (9.8) | 8771 (4.8) | <0.001 |
| Preterm birth | | 1339 (13.4) | 1613 (14.4) | 1615 (14.0) | 0.233 | 9114 (6.7) | 11022 (7.0) | 12787 (7.0) | 0.002 |
| Preeclampsia | | 580 (5.8) | 457 (4.1) | 337 (2.9) | <0.001 | 7529 (5.5) | 4808 (3.1) | 3977 (2.2) | <0.001 |
| **Neonatal Characteristics** | |  |  |  |  |  |  |  |  |
| Perinatal death | | 204 (2.0) | 196 (1.7) | 203 (1.8) | 0.139 | 1085 (0.8) | 1259 (0.8) | 1361 (0.7) | 0.046 |
| Female sex | | 4999 (49.8) | 5566 (49.6) | 5577 (48.2) | 0.018 | 66625 (48.9) | 76605 (48.8) | 89978 (48.7) | 0.437 |
| LGA | | 836 (8.4) | 903 (8.1) | 1093 (9.5) | 0.003 | 14806 (10.9) | 17592 (11.2) | 19521 (10.7) | 0.028 |
| SGA | | 1693 (17.0) | 1922 (17.2) | 1725 (15.0) | <0.001 | 11215 (8.2) | 12194 (7.8) | 14581 (8.0) | 0.016 |

*LGA* large for gestational age, *SD* standard deviation, *SES* socio-economic status, *SGA* small for gestational age

Data represented as numbers (percentages).

*The over time trends of maternal age (as a continuous variable) were assessed by simple linear regression.
