# Supplementary material for: Trends and burden of diabetes in pregnancy among Aboriginal and non-Aboriginal mothers in Western Australia, 1998–2015
Source: BMC Public Health. 2022 Feb 9;22:263. doi: 10.1186/s12889-022-12663-6 (PMC8827280; doi:10.1186/s12889-022-12663-6)
Supplement: Supplementary file 3 — Additional file 3: Figure S2. Crude prevalence of gestational diabetes among Aboriginal and non-Aboriginal pregnancies, 1998–2015. [file 12889_2022_12663_MOESM3_ESM.docx]

Figure S2: Crude prevalence of gestational diabetes among Aboriginal and non-Aboriginal pregnancies, 1998-2015

The dotted lines are the 95% confidence intervals.
