# Supplementary material for: Trends and burden of diabetes in pregnancy among Aboriginal and non-Aboriginal mothers in Western Australia, 1998–2015
Source: BMC Public Health. 2022 Feb 9;22:263. doi: 10.1186/s12889-022-12663-6 (PMC8827280; doi:10.1186/s12889-022-12663-6)
Supplement: Supplementary file 4 — Additional file 4: Figure S3. Rates of LGA over time in the Aboriginal and non-Aboriginal populations in Western Australia, 1998–2015. [file 12889_2022_12663_MOESM4_ESM.docx]

Figure S3: Rates of LGA over time in the Aboriginal and non-Aboriginal populations in Western Australia, 1998-2015

*LGA* Large for gestational age
