# Supplementary material for: Trends and burden of diabetes in pregnancy among Aboriginal and non-Aboriginal mothers in Western Australia, 1998–2015
Source: BMC Public Health. 2022 Feb 9;22:263. doi: 10.1186/s12889-022-12663-6 (PMC8827280; doi:10.1186/s12889-022-12663-6)
Supplement: Supplementary file 5 — Additional file 5: Table S2. National guidelines for screening and diagnosis of gestational diabetes during the study period (1998–2015). [file 12889_2022_12663_MOESM5_ESM.docx]

Table S2: National guidelines for screening and diagnosis of gestational diabetes during the study period (1998-2015)

| Guidelines | Screening | | | | Diagnosis | |
| --- | --- | --- | --- | --- | --- | --- |
|  | Selective or universal | Time during pregnancy | Test | Plasma glucose level thresholds | Test | Plasma glucose level thresholds |
| ADIPS 1998 (1) | Universal | 26-28 weeks | non-fasting GCT (50g) | ≥ 7.8mmol/L | OGTT (75g) | Gestational diabetes is diagnosed if the screening test is positive and one of two below criteria is met:  Fasting: ≥ 5.5 mmol/l  2 hours after OGTT: ≥8 mmol/l |
| ADIPS 2013 (2) | Universal | 24-28 weeks | OGTT (75g) | This is a single step approach. Gestational diabetes is directly diagnosed if one of the below criteria is met:  Fasting: ≥ 5.1 mmol/L  1 hour after OGTT: ≥10 mmol/L  2 hours after OGTT: ≥8.5 mmol/L | A single step approach (see screening) | |

*ADIPS* Australasian Diabetes in Pregnancy Society, *GCT* glucose challenge test, *OGTT* oral glucose tolerance test

Reference

1. Hoffman L, Nolan C, Wilson JD, Oats JJ, Simmons D. Gestational diabetes mellitus--management guidelines. The Australasian Diabetes in Pregnancy Society. Med J Aust. 1998;169(2):93-7.

2. Nankervis A, MH MR. Australasian Diabetes in Pregnancy Society (ADIPS). ADIPS consensus guidelines for the testing anddiagnosis of gestational diabetes mellitus in Australia Available online from: http://www.adips.org/downloads/ADIPSConsensusGuidelinesGDM-03.05.13VersionAcceptedFInal.pdf
